# Double Pathogenic or Likely Pathogenic Variants in Cancer Predisposition Genes in Hungarian Cancer Patients

**DOI:** 10.3390/ijms26178390

**Published:** 2025-08-29

**Authors:** Tímea Pócza, János Papp, Anikó Bozsik, Vince Kornél Grolmusz, Petra Nagy, Attila Patócs, Henriett Butz

**Affiliations:** 1 Department of Molecular Genetics and the National Tumor Biology Laboratory, National Institute of Oncology, 1122 Budapest, Hungary; 2 HUN-REN-OOI-TTK-HCEMM Oncogenomics Research Group, 1052 Budapest, Hungary; 3 Department of Laboratory Medicine, Semmelweis University, 1089 Budapest, Hungary; 4 Department of Oncology Biobank and the National Tumor Biology Laboratory, National Institute of Oncology, 1122 Budapest, Hungary

**Keywords:** cancer predisposition syndromes, gene panel, double heterozygosity, pathogen variants

## Abstract

Identification of two or more pathogenic/likely pathogenic (P/LP) variants in cancer susceptibility genes carried by the same patient have important consequences for patient management. We have limited information about the effect of double heterozygosity (DH) in cancer susceptibility genes. The prevalence of DH among Hungarian cancer patients referred to oncogenetic counselling, and comparison of their phenotypes to single variant carriers were performed. In total, 2050 patients were analysed by multigene panel sequencing. Variants of 48 established cancer predisposition genes by ACMG guidelines were evaluated. In overall, P/LP variants were found in 19.8% of cases. DH was observed in 16 cases, amount to 0.8% of all patients, and to 4.0% of positive cases. Appearance of multiple primary tumours was not associated with DH compared to non-P/LP and single P/LP carriers (*p* = 0.71 and *p* = 0.54, respectively). Within a cohort of patients referred with suspected HBOC syndrome, earlier tumour formation was observed when DH cases were compared to non-P/LP carriers (*p* = 0.01), but difference between single and DH carriers was not statistically significant (*p* = 0.19; Bonferroni corrected alpha = 0.017). Our observations provide information about the incidence of DH status among Hungarian hereditary cancer patients and suggest that DH did not increase the risk of cancer compared to individuals with single P/LP mutation.

## 1. Introduction

Hereditary cancer syndromes account for approximately 5–10% of all cancers [1,2]. These syndromes are often characterised by familial clustering of cancer-related diseases, early age of onset, or the presence of multiple primary tumours, which suggest underlying germline mutations in cancer susceptibility genes. Alterations in these genes predispose individuals to specific malignancies. *BRCA1* and *BRCA2* are among the most studied cancer susceptibility genes, significantly increasing the risk of breast, ovarian, pancreatic and prostate cancers. This condition is collectively known as hereditary breast and ovarian cancer (HBOC) syndrome. Similarly, mutations in mismatch repair (MMR) genes, including *MLH1*, *MSH2*, *MSH6*, and *PMS2*, associate with Lynch syndrome, predisposing individuals to colorectal and endometrial cancers as well as other malignancies including gastric, small bowel, biliary, urothelial, ovarian, and brain cancers [1,2,3].

Identifying pathogenic alterations in cancer susceptibility genes is critical for guiding patient management as genetic diagnoses influence surveillance strategies, preventive measures, surgical decision-making, and therapeutic approaches, such as the use of PARP inhibitors in *BRCA1/2* mutation carriers [1,3,4]. The advent of next-generation sequencing (NGS) has been revolutionised genetic diagnostics by enabling the simultaneous analysis of hundreds of genes, or even entire genomes. This has led to an increased detection of pathogenic/likely pathogenic (P/LP) variants, including patients carrying two or more P/LP variants in cancer predisposition genes [5,6].

Cases harbouring multiple germline P/LP variants in cancer susceptibility genes are commonly referred to as double heterozygosity (DH). Other terms, such as multilocus inherited neoplasia alleles syndrome (MINAS) [6,7] and transheterozygosity [8] are also used to describe this phenomenon. Although DH is rare, its frequency depends on the studied population and generally ranges between 0.2% and 1.3% [5,6,7,9,10,11,12,13,14,15,16,17]. Prior to the NGS era, only a few DH cases and gene combinations were reported, with *BRCA1-BRCA2* DH cases being the most extensively studied. Despite the growing number of identified DH cases, unique gene combinations remain rare, and the phenotypic impact of one or both variants often remains uncertain.

To date, no comprehensive study has assessed the incidence or clinical significance of DH in Hungarian cancer patients. This study aims to evaluate the phenotypes of Hungarian DH carriers and compare their clinical characteristics to those of single P/LP variant carriers.

## 2. Results

### 2.1. Cohort Characteristics and Detection Rate

The majority of consecutive patients (*n* = 2050) referred to our Comprehensive Cancer Centre had a suspicion of HBOC syndrome, which accounted for 73.0% (*n* = 1496), followed by patients with Lynch syndrome-related tumours or polyposis, which accounted for 13.1% (*n* = 268). Further, 7.1% (*n* = 146) of the patients had endocrine-system originated cancer, and 4.1% (*n* = 84) of cases had other tumour types (e.g., melanoma, renal, bladder, liver cancer, etc.). The remaining 2.7% (*n* = 56) of the cohort comprised individuals who were not affected by a malignant disorder, but for whom the rationale for genetic testing was based on the family history. Demographic and clinical parameters of patient’s subgroups are summarised in Table 1. Females predominated in the group of suspected HBOC cases, accounting for 95.5% (*n* = 1428) and in the group of unaffected patients, accounting for 100% (*n* = 56; 100%) of cases. Men represented 30.2% (*n* = 81) of the Lynch/polyposis group, 41.1% (*n* = 60) of and 46.4% (*n* = 39) of patients with endocrine and other tumours, respectively. The mean age of disease onset differed between these groups. In the HBOC and Lynch/polyposis suspect cohorts, the mean age of onset was 49.5 and 51.9 years, respectively, whereas patients with endocrine manifestations and other tumours developed symptoms at an earlier age, 41.2 and 41.9 years, respectively.

Pathogenic or likely pathogenic (P/LP) variants were identified in 19.8% (*n* = 405) of all cases, with the highest prevalence observed in patients with suspected Lynch/polyposis syndrome (27.2%). The detection rate was 18.5% in patients with suspected HBOC syndrome, 21.2% in those with endocrine tumours, 21.4% in patients with other tumour types, and 10.7% in unaffected individuals.

**Figure 1:**
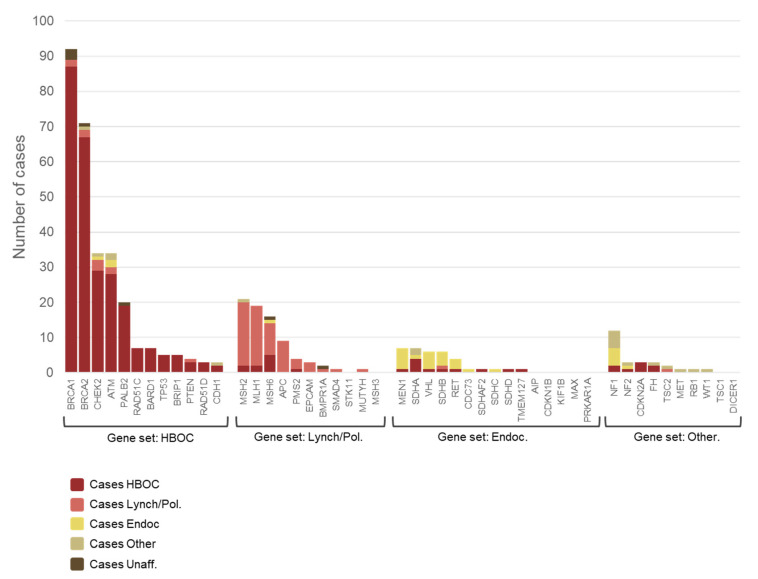
Number of positive cases according to affected genes, categorised by phenotype. Genes are grouped based on the syndrome they are associated with.

P/LP variants were predominantly found in genes expected from the phenotype (Figure 1). Namely, 91.3% of suspected HBOC cases, 84.9% of patients with suspected Lynch/polyposis syndrome and 67.7% of patients with endocrine manifestations have variants in genes corresponding to the patient’s phenotype. The relatively lower rate in the endocrine group may be due to a variable and mixed group of patients. The P/LP variant ratio was the highest in the group of Lynch/polyposis suspects and the lowest in the unaffected cases.

### 2.2. Double Heterozygosity

Sixteen (0.8% of all patients) individuals were identified carrying two or more P/LP variants among 2050 patients suspected of hereditary cancer. Of these, 13 patients had suspected HBOC syndrome (0.9% within suspected HBOC cases): 11 presented with breast cancer, one with both breast and endometrial cancer, and one with prostate cancer. One-one case was identified in the Lynch/polyposis, other, and unaffected patient cohorts, giving frequencies of 0.4%, 1.2%, and 1.8%, respectively (Table 1). The clinicopathological characteristics and P/LP variants of these double-hit cases are detailed in Table 2A,B.

In nine cases, two P/LP variants were found in genes associated with HBOC. Five cases exhibited combinations of HBOC and endocrine or Lynch syndrome genes (e.g., *SDHAF2*, *SDHA*, *MSH6*). One patient carried P/LP variants in *MSH*2 (a Lynch syndrome gene) and *SDHB* (associated with paraganglioma and pheochromocytoma). One patient carried three P/LP variants, two in genes associated with HBOC and one associated with endocrine syndromes (Table 2A,B).

**Figure 2:**
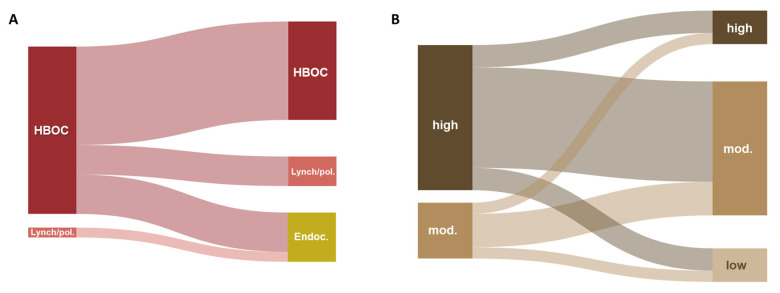
Illustration of combinations of cancer susceptibility genes in DH patients, categorised by the associated syndrome (**A**) and by gene penetrance (**B**). The first column includes genes with variants most relevant to the patient’s phenotype. In cases with three P/LP variants in the same patient, all possible gene pairs were included. Nodes represent the syndrome associated with each gene (**A**) or the level of penetrance of genes (**B**). High penetrance genes: *BRCA1*, *BRCA2*, *CDH1*, *PALB2*, *SDHAF2*; Moderate penetrance genes: *ATM*, *BARD1*, *BRIP1*, *CHEK2*, *MSH6*, *SDHB.*; Low penetrance genes: *SDHA*.

Two patients carried both variants in high-penetrance genes, while 11 had one high- and one/or two moderate/low-penetrance variant(s), and three had both variants in moderate/low-penetrance genes (Figure 2A,B). Genes were classified as high, moderate and low risk genes based on their penetrance data, focusing on the highest risk regardless of the type of tumour, using former published data and data from National Comprehensive Cancer Network (NCCN) guidelines [18,19,20,21,22].

Of the 10 cases with double P/LP variants in HBOC genes, eight had breast cancer, one had prostate cancer, and one had a fumarate hydratase (FH)-deficient leiomyoma with a family history of breast cancer. In three cases, *BRCA1/2* variants co-occurred with *MSH6* variants. In four cases, *SDHA* and *SDHAF2* variants were identified together with HBOC genes. One of these had three P/LP variants (*BRCA1*, *ATM* and *SDHA)* (case #12; Table 2B). Another case (case #2) had *MSH2* and *SDHB* variants, which predispose to hereditary pheochromocytoma-paraganglioma syndrome. Of the 16 DH cases, two (case #4 and #15) involved P/LP variants within the same gene (*CHEK2*). The cis-trans positioning was not determined, but familial data suggested a trans configuration for one of them (Supplementary Figure S1).

In cases carrying P/LP variants in two different syndrome genes, the phenotype typically reflected the effect of one variant. In one case, neither of the found P/LP variants explained the phenotype: a patient with FH-deficient leiomyoma had P/LP variants in *CDH1* and *ATM* (case #16). An unaffected patient carrying a *BRCA1* and *MSH6* variants had a family history of breast cancer (case #5). Case #10 with P/LP variants in *CHEK2* and *SDHAF2* variants had a mixed phenotype with endometrial and breast cancer.

Two of the DH cases exhibited second or third solid malignancies. The frequency of multiple solid tumours among DH cases (12.5%) was not elevated compared to non-carriers (11.7%; *p* = 0.71) and single-variant carriers (21.9%; *p* = 0.54) analysed by Fisher’s exact test. No significant difference was found when restricted to HBOC-suspected cases (*p* = 0.66 and *p* = 0.74 compared to non- and single-variant carriers, respectively).

**Figure 3:**
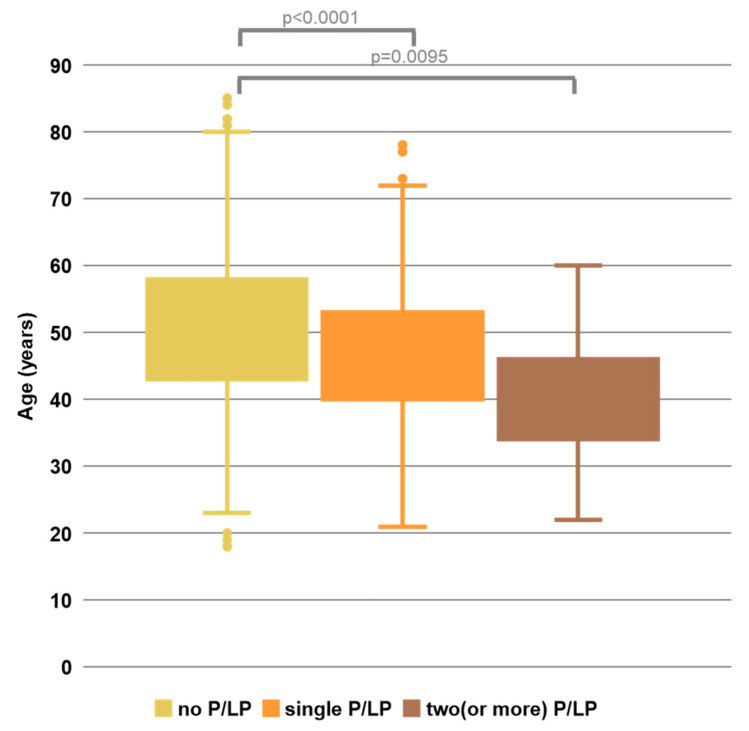
Boxplot showing the age of disease onset among patients suspected of HBOC syndrome predisposition. Patients were grouped based on the number of P/LP variants carried: none, one, or two (or more).

To evaluate whether DH status influences the age of tumour onset, HBOC-suspected patients were analysed. The mean ages at diagnosis were 50.2, 46.8, and 40.7 years for non-carriers, single-variant carriers, and DH cases, respectively. While both single-variant and DH cases had significantly lower ages than non-carriers, the difference between single-variant and DH cases was not statistically significant analysed by the Kruskal–Wallis analysis of variance with Dunn’s post hoc test (*p* = 0.19; Bonferroni-corrected α = 0.017). However, DH cases tended to have a lower mean age (40.7 years vs. 46.8 years; Figure 3).

## 3. Discussion

The introduction of NGS has revolutionised genetic analysis, enabling its integration into clinical practice. NGS provides a cost-effective method for generating vast amounts of genetic data, including information about genes not directly related to the patient’s phenotype. Consequently, the detection of complex genotypes with multiple P/LP variants is increasing. However, predicting the phenotypic effects of harbouring two or more P/LP variants in cancer-predisposing genes remains a challenge [5,6].

The *BRCA1-BRCA2* combination has been the most extensively studied. Some reports indicated that double-hit carriers develop cancer at an earlier age compared to single P/LP carriers [11,13,23]. However, analyses based on the Consortium of Investigators of Modifiers of *BRCA1/2* (CIMBA dataset) have suggested that DH carriers exhibit a phenotype similar to *BRCA1* carriers alone [8]. Other studies found no significant difference in disease severity between DH and single-variant carriers, particularly in breast cancer patients [14,24]. Former studies performed systematic reviews to describe the genetic architecture and phenotypic consequences of DH/MINAS cases. In a former study, 82 cases were collected with co-occurring P/LP variants in 17 hereditary cancer genes [7]. The most frequently reported combinations were *BRCA1*/*BRCA2*, *BRCA2*/*TP53*, *BRCA1*/*MLH1*, and *APC*/*MLH1*, reflecting the relevance of the most commonly screened genes for major hereditary cancer syndromes, as well as the presence of founder variants in certain populations. A later update review [6] analysed 385 patients with two or more P/LP variants. At least one P/LP variant in the *BRCA1* and/or *BRCA2* genes was identified in most cases (74.6%). The ratio of multiple primary tumours was 28.1% in this cohort of patients, and 15% of patients showed an atypical tumour phenotype (tumours not related to any of the disrupted genes). The authors concluded that the clinical phenotype was rather an independent effect of two (or more) P/LP variants; however, some cases with unusual tumour phenotypes raise the possibility of complex interaction [6]. In a retrospective study, several DH cases were identified, showing a younger age of onset in *ATM*/*CHEK2* and double *CHEK2* carriers [25]. A more recent review investigated correlations between the DH status and clinical characteristics (*n* = 413). Based on their results, DH carriers tend to have more malignancies and an early onset of the first cancer [26].

The controversial conclusions of studies dealing with multiple pathogenic variants among cancer patients may be due to the different genetic architecture of DH cases in the studied cohorts, different founder mutations in some populations, the slightly different gene panels used and the different assignment of PVs. For example, *MUTYH* heterozygous variants are often referred to as pathogenic variants despite their autosomal recessive inheritance [15].

This is the first study assessing the prevalence and phenotypic features of DH cases in a Hungarian cohort of patients suspected of hereditary cancer predisposition. Previous studies using large gene panels reported DH frequencies of 0.2–1.3% in all patients and 1.8–6.2% of P/LP variants. In our cohort, DH cases comprised 0.8% of all patients and 4.1% of P/LP carriers, aligning with earlier findings [5,12,13,14,15,16,17]. Of the P/LP positive cases, DH carriers were more prevalent among HBOC cases (0.9%) compared to Lynch/polyposis cases (0.4%).

Overall, in our cohort, P/LP variants were most frequently detected in patients with suspected Lynch/polyposis syndrome (27.2%). The prevalence was 18.5% in patients with suspected HBOC syndrome, and slightly above 20% in those with endocrine tumours (21.2%) and other tumour types (21.4%). As expected, the lowest P/LP detection rate was observed in the group of unaffected individuals (10.7%). The detection rates of P/LP variants in patients with suspected HBOC and Lynch/polyposis syndromes are consistent with our previous findings [27,28]. Other studies have also reported similar frequencies of P/LP variants using multigene panel testing, although rates vary depending on cancer type and the panel applied [20,29,30].

Our analysis found no evidence that DH contributes to more primary solid tumour development or significantly influences the clinical phenotype, in agreement with other studies [14]. Similarly, our results showed no earlier tumour onset among DH carriers compared to single-variant carriers, aligning with findings by other researchers [10,16,31]. While our DH carriers had a lower mean age at diagnosis, the difference was not statistically significant, likely due to the small sample size.

Some studies suggested a higher rate of multiple primary tumours among DH carriers (28%) [6] and found that DH status was associated with multiple primary cancers [26]. In contrast, our data showed that the rate of multiple independent (non-syndromic) malignancies in our DH cohort was 12.5%. This might be related to the patient group studied. Our study included consecutive cancer patients referred for genetic counselling.

Interestingly, despite *BRCA1* and *BRCA2* accounting for most pathogenic variants in our study, only one case had DH of *BRCA1-BRCA2*. Moreover, high-penetrance cancer susceptibility gene combinations were rare (2/16 cases: *BRCA1-BRCA2* and *BRCA2-CDH1*). Seven of the 16 DH cases involved secondary findings in genes, such as *MSH6* or *SDHx* genes, not associated with the phenotype and without associated tumour types observed during follow-up. The phenotypes of DH cases in our cohort seem to support that the effect of two P/LP variants on cancer risk is probably independent rather than synergistic, as suggested by former studies [5,6].

In conclusion, DH carriers did not show an increased risk of earlier tumour onset or multiple primary tumours compared to patients with only one P/LP variant in tumour susceptibility genes. DH remains rare among Hungarian cancer patients, with approximately 1 in 25 P/LP carriers harbouring an additional variant. While rare, DH has significant implications for offspring and family screening, with a 75% chance of inheriting one or two P/LP variants, compared to 50% for single carriers. Our findings suggest that DH patients do not require stricter surveillance than recommended for their most severe P/LP variant. However, specific gene combinations may warrant tighter protocols, as suggested by some studies [25,32,33]. Limitations of our study include the small sample size and lack of long-term follow-up data. In our workflow, self-reported family histories were collected during pre-test counselling, which may contain inaccuracies and represent a limitation of our study [34]. However, in our practice, sending a questionnaire in advance regarding medical and family history improved accuracy. Our previous experience showed that families of P/LP variant carriers present with significantly more syndrome-specific tumours compared to probands without a genetic predisposition [27]. Therefore, we consider our approach to adequately reflect the clinical situation. Further systematic studies are needed to clarify the clinical relevance of DH and its impact on cancer predisposition and management.

## 4. Materials and Methods

### 4.1. Patients

Two thousand fifty consecutive, unrelated patients referred to the Department of Molecular Genetics of the National Institute of Oncology (Budapest, Hungary) for possible hereditary tumour syndrome between March 2021 and August 2023 were included in the study. In accordance with Hungarian legislation, all patients were provided with clinical genetic (pre-test) counselling, during which all aspects of the molecular genetic tests were discussed (https://kollegium.okfo.gov.hu/iranyelvek (accessed on 25 August 2025)). During genetic counselling, all patients provided written informed consent to undergo genetic testing and participate in related research. Relevant medical history and family data were collected. The indication for genetic testing was assessed according to international guidelines, including the NCCN Guidelines Version 2.2024: Genetic/Familial High-Risk Assessment: Breast, Ovarian, and Pancreatic, the NCCN Guidelines Version 1.2024: Genetic/Familial High-Risk Assessment: Colorectal, Endometrial, and Gastric, as well as established clinical guidelines for endocrine tumours [35]. Following the completion of molecular genetic testing, the results were presented to the patients during post-test counselling. Being a comprehensive centre, patients with potential hereditary predisposition for several types of cancer, including breast, ovarian and colorectal cancer, as well as endocrine or other rare tumours, have been evaluated. The study was approved by the Scientific and Research Committee of the Medical Research Council of the Ministry of Health, Hungary (ETT-TUKEB 53720-4/2019/EÜIG, ETT-TUKEB 4457/2012/EKU).

### 4.2. Genetic Analysis

Peripheral blood samples were collected from patients, and genomic DNA was isolated using Gentra Puregene Blood Kit (Cat No.: 158389, Qiagen, Hilden, Germany) according to the manufacturer’s instructions. For genetic testing, the TruSight Hereditary Cancer Panel (#20029551, Illumina, San Diego, CA, USA) was employed, which contains 113 genes that are predisposing to various cancers. Library construction and next-generation sequencing were performed on an Illumina MiSeq or NextSeq 550Dx platforms, according to the manufacturer’s instructions. Data were analysed by DRAGEN Enrichment pipeline (Version 4.0.3, Illumina, San Diego, CA, USA). Sequencing reads were aligned to the human GRCh38 genome reference. Variants were filtered as follows: 3% threshold for population allele frequency (data imported from gnomAD v2.1.1), the region of interest covered the exonic target regions, which were extended to overreach exon/intron boundaries by 30 base pairs. DRAGEN pipeline was used for small nucleotide variant, copy number variant (CNV) and structural variant (SV) calling. Variants with a total read depth was above 10, and variant allele frequency was above 20% were further interpreted. For CNVs and SVs calls, highly recurrent variants (>1% in our examined cohort) were omitted.

In addition to the DRAGEN pipeline, we also used the VarSeq CNV algorithm (v2.6.2 Golden Helix, Bozeman, Montana, USA) for CNV calling. Calls were retained when *p* < 0.05 and the Z-score  ≤  −3 or Z-score  ≥  3, and normalised read depth < 0.7 or >1.2 for heterozygous deletions and duplications, respectively. Variants were annotated by BaseSpace Variant Interpreter (Illumina, San Diego, CA, USA) and VarSeq (Golden Helix, Bozeman, MT, USA).

For variant classification, the American College of Medical Genetics and Genomics (ACMG) [36] guideline was used, supplemented by the information deposited in NBCI ClinVar (https://www.ncbi.nlm.nih.gov/clinvar/ (accessed between 1 September 2021–12 January 2025)), Franklin (https://franklin.genoox.com/clinical-db/home (accessed between 1 September 2021–12 January 2025)) and Varsome (https://varsome.com/) databases. Of the 113 genes of the applied panel, only a set of 48 selected genes was evaluated based on the NCCN (2024) and ACMG [36] guidelines for HBOC (NCCN Guidelines Version 2.2024, Genetic/Familial High-Risk Assessment: Breast, Ovarian, and Pancreatic) and colorectal, endometrial, and gastric cancers (NCCN Guidelines Version 1.2024, Genetic/Familial High-Risk Assessment: Colorectal, Endometrial, and Gastric), and also well-established genes predispose to endocrine and other tumour syndromes (Supplementary Table S1) [1,35,37,38]. Regarding genes with recessive inheritance, e.g., *MUTYH* and *MSH3*, only biallelic P/LP variants were considered as one hit. For *EPCAM* variants, only deletions of the 3’ end were accepted as pathogenic. Low penetrance *CHEK2* variants (p.I157T, p.T476M and p.S428F) were excluded from our analysis, due to their inconsistent interpretation and lack of clinical consequence [39]. Due to similar reasons, *APC* p.I1307K was not considered as a P/LP variant [40,41].

All P/LP variants were validated by the “gold standard” Sanger sequencing for single-nucleotide variants (SNVs) and for small insertions/deletions, and by multiplex ligation-dependent probe amplification (MLPA; SALSA MLPA probe mixes for appropriate genes, probe sets are listed in Supplementary Table S1, MRC-Holland, Amsterdam, The Netherlands) for large-scale CNV changes. The Illumina Hereditary Cancer Panel we used is specifically designed to avoid interference from pseudogenes. Target enrichment is achieved by hybridisation-based capture with unique probes that selectively bind to the canonical genes, thereby minimising pseudogene amplification. DRAGEN (Illumina) pipeline applies stringent alignment algorithms to uniquely map reads to the canonical locus. In addition, all pathogenic and likely pathogenic variants were validated by Sanger sequencing. During primer design, care was taken to avoid products derived from pseudogenes, providing locus-specific confirmation.

### 4.3. Statistical Analyses

To evaluate whether DH has any disadvantages in the development of primary solid tumours, we examined whether there is an earlier age of disease onset or a trend to form multiple primary solid tumours in the probands. The occurrence of multiple primary solid tumours was compared between cases of DH and those with a single or non-P/LP variant carriers by Fisher’s exact test. A comparison of the age of disease onset between DH cases and single or non-P/LP cases was conducted exclusively within the patient cohort referred for HBOC suspicion to ensure the homogeneity of the samples, given that the mean age of different disease groups varies significantly. The number of DH cases in the other groups was insufficient to perform statistical analysis. Statistical analysis was performed using GraphPad Prism (v.8.0.1) software. A Kruskal–Wallis test with Dunn’s post hoc test was employed to ascertain whether there were any significant differences in the age of disease onset between the following groups: no variant carriers, single variant carriers and two (or more) variant carriers among HBOC-suspected patients. Bonferroni correction was applied for multiple comparisons, and *p*-values < 0.017 were accepted as statistically significant.

## Figures and Tables

**Table 1:** Demographic characteristics, prevalence of P/LP variants and ratio of DH status among patients grouped by suspected tumour syndrome. n.a.: not applicable.

	HBOC	Lynch + Pol.	Endocrine	Other	Unaffected	Sum
Case number	1496	268	146	84	56	2050
%	73.0	13.1	7.1	4.1	2.7	100
Gender						
Female (number)	1428	187	86	45	56	1802
%	95.5	69.8	58.9	53.6	100.0	87.9
Male (number)	68	81	60	39	0	248
%	4.5	30.2	41.1	46.4	0.0	12.1
Mean age (years)	49.5	51.9	41.2	41.9	n.a.	
SD	11.5	15.4	18.3	19.4	n.a.	
Median age (years)	48	54	42	44	n.a.	48
min	18	5	0 ^a^	0 ^b^	n.a.	0 ^b^
max	85	78	83	80	n.a.	85
Muliple primaries	203	49	5	22	n.a.	279
%	13.6	18.3	3.4	26.2	n.a.	13.6
Number of P/LP cases	277	73	31	18	6	405
%	18.5	27.2	21.2	21.4	10.7	19.8
Number of DH cases	13	1	0	1	1	16
%	0.9	0.4	0.0	1.2	1.8	0.8

^a^ age: 6 months. ^b^ age: 1 month.

**Table 2:** (**A**) Clinical characteristics of DH patients. (**B**) Variant description and genomic consequences in DH cases. Patients’ order is the same as in (**A**).

(**A**)												
**#**	**Sex**	**Suspected Sy**	**Tumour_1**	**Age**	**Tumour_2**	**Age**	**MPs**	**Tumour Histology**		**Family History**		
1	FEM	HBOC	br	44				IDC; ER +; PR +; Her2 -; Ki67 80%		br (52 + 56); br (58)		
2	FEM	Lynch/Polyposis	end	36				endometrioid cc with squamous differentiation		Lung (?); cr (43); st (37); st (53); br (60) + ov (60)		
3	FEM	HBOC	br	22				IDC; ER -; PR -; Her2 -; Ki67 70%		Skin (45); br (77); cr (70)		
4	FEM	HBOC	br	37				IDC; ER +; PR -; Her2 +; Ki67 80%		br (58); br (59 + 65); st (49); st (86)		
5	FEM	unaff	unaff					unaffected		Br (42); br (?)		
6	FEM	HBOC	br	45	NHL	50		IDC; ER -; PR -; Her2 -; Ki67 ?%		cr (60); br (57); gb (?); cr (?); ut (51) + ov (51)		
7	FEM	HBOC	br	47				IDC; ER +; PR +; Her2 -; Ki67 40–50%		br (44)		
8	FEM	HBOC	br	30				IDC; ER +; PR +; Her2 ?; Ki67 20%		br (53); br (66); cr (58); cr (50)		
9	MAL	HBOC	prostate	60				adenocc.		br (49); nb (2)		
10	FEM	HBOC	end	54	breast	66	y	adenocc.; LCIS + ILC; ER +; PR +; Her2 -; Ki67 1–2%		br (42); brain (43); ren (?); brain (?); st (?)		
11	FEM	HBOC	br	35				IDC; ER -; PR -; Her2 -; Ki67 50%		br (68); lung (54); bone (?)		
12	FEM	HBOC	br	42				IDC + DCIS; ER -; PR -; Her2 +; Ki67 10%		br (?); lung (?)		
13	FEM	HBOC	br	33				IDC; ER -; PR -; Her2 -; Ki67 80%		br (55)		
14	FEM	HBOC	br (bilat)	37			y	ILC; ER +; PR +; Her2 +; Ki67 10–15%		pros (46); panc (46); liver (46)		
15	FEM	HBOC	br	43				IDC; ER +; PR +; Her2 -; Ki67 10%		Cervix (44)		
16	FEM	other	ut (leiomyoma)	32				FH deficient ut leiomyoma		br (48); br (66); larynx (52)		
(**B**)												
**#**	**Gene_1**	**HGVS** **Transcript_1**		**HGVS** **Protein_1**			**Gene_2**	**HGVS** **Transcript_2**	**HGVS** **Protein_2**	**Gene_3**	**HGVS Transcript_3**	**HGVS** **Protein_3**
1	*ATM*	NM_000051.4:c.6679C>T		NP_000042.3:p.(Arg2227Cys)			*BARD1*	NM_000465.4:c.1932_1933del	NP_000456.2:p.(Cys645Ter)			
2	*MSH2*	NM_000251.3:c.2068C>T		NP_000242.1:p.(Gln690Ter)			*SDHB*	NM_003000.3:c.148G>T	NP_002991.2:p.(Asp50Tyr)			
3	*BRCA1*	NM_007294.4:c.5266dup		NP_009225.1:p.(Gln1756ProfsTer74)			*MSH6*	NM_000179.3:c.3261del	NP_000170.1:p.(Phe1088SerfsTer2)			
4	*CHEK2*	NM_007194.4: del(exon 9–10)		NP_009125.1:p.?			*CHEK2*	NM_007194.4:c.499G>A	NP_009125.1:p.(Gly167Arg)			
5	*BRCA1*	NM_007294.4:c.181T>G		NP_009225.1:p.(Cys61Gly)			*MSH6*	NM_000179.3:c.3379_3438+5del	NP_000170.1:p.?			
6	*PALB2*	NM_024675.4: del(exon 11)		NP_078951.2:p.?			*SDHA*	NM_004168.4:c.91C>T	NP_004159.2:p.(Arg31Ter)			
7	*BRCA2*	NM_000059.4:c.5682C>G		NP_000050.3:p.(Tyr1894Ter)			*BRIP1*	NM_032043.3:c.3525dup	NP_114432.2:p.(Ile1176TyrfsTer13)			
8	*BRCA2*	NM_000059.4:c.8249_8251del		NP_000050.3:p.(Lys2750del)			*ATM*	NM_000051.4:c.1564_1565del	NP_000042.3:p.(Glu522IlefsTer43)			
9	*BRCA1*	NM_007294.4:c.181T>G		NP_009225.1:p.(Cys61Gly)			*BRCA2*	NM_000059.4:c.6295A>T	NP_000050.3:p.(Arg2099Ter)			
10	*CHEK2*	NM_007194.4:c.1100del		NP_009125.1:p.(Thr367MetfsTer15)			*SDHAF2*	NM_017841.4:c.446_447del	NP_060311.1:p.(Lys149ArgfsTer10)			
11	*BRCA2*	NM_000059.4:c.5073dup		NP_000050.3:p.(Trp1692MetfsTer3)			*MSH6*	NM_000179.3:c.3379_3438+5del	NP_000170.1:p.?			
12	*BRCA1*	NM_007294.4:c.181T>G		NP_009225.1:p.(Cys61Gly)			*ATM*	NM_000051.4:c.7630-2A>C	NP_000042.3:p.?	*SDHA*	del (whole gene)	NP_004159.2:p.?
13	*BRCA1*	NM_007294.4:c.181T>G		NP_009225.1:p.(Cys61Gly)			*BRIP1*	NM_032043.3:c.1741C>T	NP_114432.2:p.(Arg581Ter)			
14	*BRCA2*	NM_000059.4:c.5645C>A		NP_000050.3:p.(Ser1882Ter)			*CDH1*	NM_004360.5:c.1711+5G>A	NP_004351.1:p.?			
15	*CHEK2*	NM_007194.4:c.1337del		NP_009125.1:p.(Asn446ThrfsTer23)			*CHEK2*	NM_007194.4:c.277del	NP_009125.1:p.(Trp93GlyfsTer17)			
16	*ATM*	NM_000051.4:c.381del		NP_000042.3:p.(Val128Ter)			*CDH1*	NM_004360.5:c.1063_1066dup	NP_004351.1:p.(Ser356IlefsTer13)			

Abbreviations: cc carcinoma; cr colorectal; br breast; ccRCC clear cell renal cell carcinoma; end endometrial carcinoma; ov ovarial; nb neuroblastoma; panc pancreas; pros prostate; PTC papillary thyroid carcinoma; st stomach; ut uterus. '?' indicates that the age at tumour onset is unknown.

## Data Availability

All data in the study are included in the article, further inquiries can be directed to the corresponding author.

## Supplementary Materials

The following supporting information can be downloaded at: https://www.mdpi.com/article/10.3390/ijms26178390/s1.
