# Supplementary material for: Double Pathogenic or Likely Pathogenic Variants in Cancer Predisposition Genes in Hungarian Cancer Patients
Source: Int J Mol Sci. 2025 Aug 29;26(17):8390. doi: 10.3390/ijms26178390 (PMC12428717; doi:10.3390/ijms26178390)
Supplement: Supplementary file 1 [file ijms-26-08390-s001.zip › ijms-3835117-supplementary.pdf]

Figure S1.

The Pedigrees of *CHEK2-CHEK2* DH Carriers.

For proband #15, only one relative was available for testing and was found to carry only one of the two *CHEK2* P/LP variants. In case #4, no other family members were available for genetic testing. Tested individuals are marked with red circles

Pedigree of Case #4

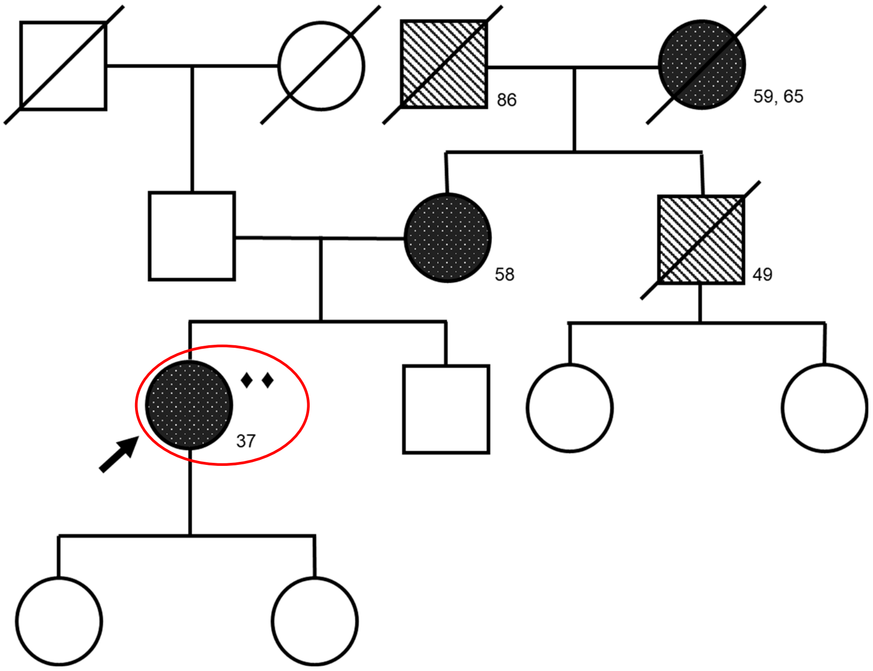

Pedigree of Case #15

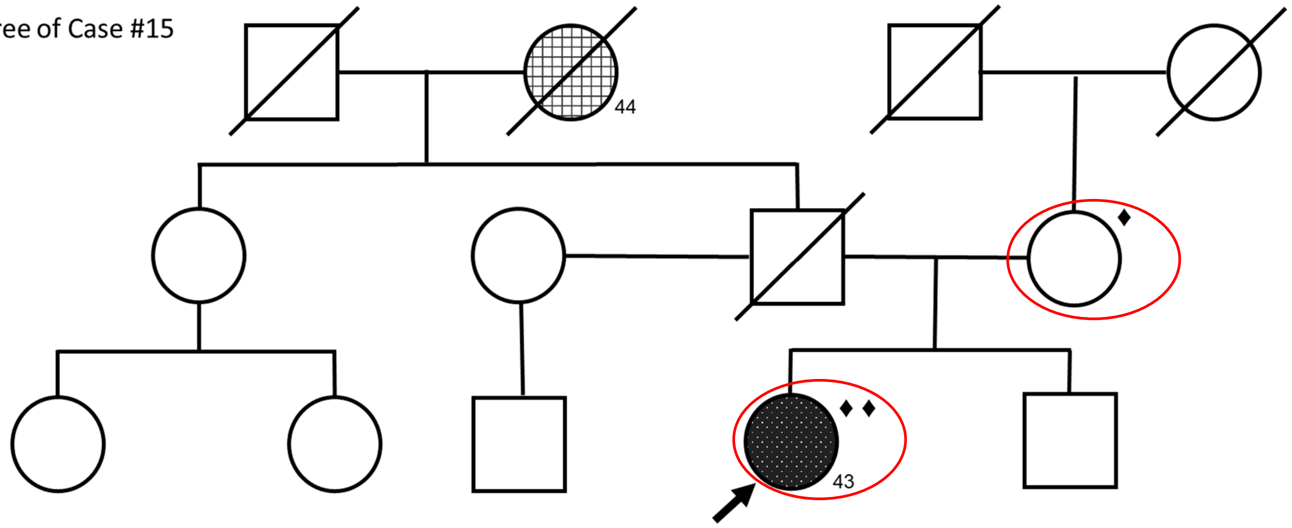

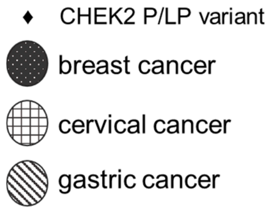

Table S1.

list of evaluated genes and applied MLPA probe sets.

#	Gene name	NCBI Reference Sequence	MPLA probe set
1	AIP	NM_003977.4	
2	APC	NM_000038.6	P043-E2
3	ATM	NM_000051.4	P041-B1, P042-B2 *
4	BARD1	NM_000465.4	P489-A1*
5	BMPR1A	NM_004329.3	P158-D1
6	BRCA1	NM_007294.4	P002-D1, P293-B1 *
7	BRCA2	NM_000059.4	P045-D1 *
8	BRIP1	NM_032043.3	P240-A5
9	CDC73	NM_024529.5	
10	CDH1	NM_004360.5	P083-D2
11	CDKN1B	NM_004064.5	
12	CDKN2A	NM_000077.5	
13	CHEK2	NM_007194.4	P190-D1 *
14	DICER1	NM_177438.3	
15	EPCAM	NM_002354.3	P072-D1 *
16	FH	NM_000143.4	
17	KIF1B	NM_001365951.3	
18	MAX	NM_002382.5	P429-C1
19	MEN1	NM_001370259.2	P017-D1
20	MET	NM_000245.4	
21	MLH1	NM_000249.4	P003-D1 *
22	MSH2	NM_000251.3	P003-D1 *
23	MSH3 biallelic	NM_002439.5	
24	MSH6	NM_000179.3	P072-D1 *
25	MUTYH biallelic	NM_001048174.2	P072-D1
26	NF1	NM_001042492.3	P081-D1, P082-D2
27	NF2	NM_000268.4	
28	PALB2	NM_024675.4	P260-C1 *
29	PMS2	NM_000535.7	P008-C1 *
30	PRKAR1A	NM_002734.5	P481-A1
31	PTEN	NM_000314.8	P225-E1
32	RAD51C	NM_058216.3	P260-C1 *
33	RAD51D	NM_002878.4	P260-C1
34	RB1	NM_000321.3	P047-E2 *
35	RET	NM_020975.6	P169-C2
36	SDHA	NM_004168.4	P429-C1 *
37	SDHAF2	NM_017841.4	P226-D1
38	SDHB	NM_003000.3	P226-D1 *
39	SDHC	NM_003001.5	P226-D1
40	SDHD	NM_003002.4	P226-D1 *
41	SMAD4	NM_005359.6	P158-D1
42	STK11	NM_000455.5	P158-C2
43	TMEM127	NM_017849.4	P429-C1
44	TP53	NM_000546.6	P056-D1
45	TSC1	NM_000368.5	
46	TSC2	NM_000548.5	P046-D1
47	VHL	NM_000551.4	P016-C2 *
48	WT1	NM_024426.6	P118-C3 *

* with positive results
